# Strengthening the Engagement of Provinces in Health Workforce Planning and Management: A Case Study From Lao PDR

**DOI:** 10.2188/jea.JE20160094

**Published:** 2016-07-05

**Authors:** Khampasong Theppanya, Outavong Phathammavong, Arie Rotem

**Affiliations:** 1 Department of Health Personnel, Ministry of Health, Vientiane Capital, Lao PDR; 2 Health Sector Support Programme Phase II (LAO/027 Project), Luxembourg Agency for Development Cooperation, Vientiane Capital, Lao PDR; 3 University of New South Wales, New South Wales, Australia

**Keywords:** health workforce, health staff projection, sub-national planning, human resource management, health staff productivity

## Abstract

The purpose of this health workforce plan is to provide guidance for the staffing of the Bolikhamxay. Province health services and the training of health service personnel to the year 2020. It must be stressed, however, that this plan is in its first iteration and does not provide all the solutions. Rather, it identifies issues that need to be further investigated and resolved at the local level. For example, the provincial health department (PHD) will need to further investigate the reasons for the significant variability in the utilization of services in different facilities and in the different ratios of staff in relation to the activities performed. The accuracy of the data must be validated and specific interventions must be determined. For Bolikhamxay, particular attention by PHD and district health authorities should be given to the following issues identified in the analysis:

• Shortage of clinical staff, particularly in the age group 30 to 40 years old, to provide supervision, guidance, and support for junior staff in coming years;
• The existence of health centers with less than minimum staffing level (<3), including a midwife and/or staff capable of properly addressing emergencies with particular reference to maternal and child health.
• The median number of activities per staff per year is around 470 (Nakoun/Bolikhan), which means that, on average, a health worker will participate in fewer than two activities per day. The situation in some district hospitals and most health centers is even worse, with an annual average numbetbl06r of activities per staff of only 163, which means that, on average, one staff participates in one activity every 3 days, hardly enough to maintain skills and justify deployment.
• This low level of staff activity raises questions about the need for further increase of staff supply to health centers and districts unless effective interventions are implemented to increase the demand and utilization of services in these facilities.
• It is also necessary to document all relevant activities, including outreach activities and home visits, in order to give appropriate weight in the calculation of utilization and productivity.
• Development of the provincial health workforce development plan requires validated human resources for health information and engagement of local health authorities, as well as strong collaboration with the national authorities and development partners, to ensure adequate support and resourcing.

## BACKGROUND

Shortage, maldistribution, and misutilization of qualified health professional staff, including mid- and high-level medical staff, nurses, and midwifes (the World Health Organization [WHO] definition), particularly at the provincial and district levels, poses a major challenge for the Lao People’s Democratic Republic (PDR) health system. According to annual statistics of the Department of Health Personnel (DHP) of the Lao Ministry of Health (MOH) in 2015, there were approximately 14 qualified health professionals per 10 000 population working at public health facilities nationwide, which represents a significant increase from approximately 5 staff per 10 000 population in 2005. Moreover, inequity of staff distribution between urban and rural/hard-to-reach areas, improper skill mix (especially at district and health center [HC] levels), and weak supervision mechanisms by qualified upper-level staff, resulted in low productivity of health staff in local settings.^1^ Improvement of human resources for health, therefore, is among the top national health reform priorities of the government of Lao PDR as a crucial factor to achieve its goal of universal health coverage (UHC) by 2025.^2^

In Laos, there is one University of Health Sciences where bachelors in medicine and nursing are offered, and there are three provincial public health colleges providing diplomas in nursing and primary health care. In total, these institutes produce more than 1000 graduates annually.^3^ With this level of production, Laos could achieve sufficient numbers of qualified health staff by population as defined by the WHO within a few years.^4^ However, as noted by both national and international observers, the graduates of these training institutions are often not sufficiently competent and professionally prepared to deliver quality services. Further well-documented capacity building, including reform of curriculum, training of academic staff, improvement of facilities and training settings (in hospitals and the main campuses), as well as leadership and enabling policies that promote excellence and provide adequate incentives, are required.

To address these human resources for health (HRH) challenges, the MOH developed the Health Personnel Development Strategy by 2020, which was endorsed by the government in 2010. The strategy included a projection and justification for the number and mix of staff needed by 2020 based on analysis of the functions performed at each level of the health system. Given the national priority of addressing maternal and child health issues, great attention was given to production of midwives and strengthening skilled birth attendant (SBA) capabilities. In projecting the total number of doctors required, for example, it was noted that, the MOH needs to recruit an average of approximately 1041 new medical graduates annually for 10 years to achieve the agreed HRH targets.^5^ In keeping with this strategy, the civil servant quota for the MOH was increased approximately 10-fold (to 4000) in fiscal year 2014–2015. Improving the quality of staff performance remains a major challenge. It has been noted that the health sector institutional capacity for regulation, enforcement, and transforming policy into effective implementation is still limited, so allocation and distribution of staff to provinces is often inappropriate, leading to deficiencies in service provision. Furthermore, HRH planning beyond the macro level, including detailed analysis of the local context, is essential to engage the provinces that manage local hospitals and health facilities and deploy health workers (HWs).

The national HRH strategy is intended to set national priorities, promote enabling policies in consultation with other sectors, and provide detailed estimates of the overall production and recruitment requirements based on estimates of the average staffing requirement to perform the essential functions at each level of the system. The availability of a national framework and standards enables estimation of the overall staff requirements and associated costs, leading to better allocation of resources and effective advocacy for needed support. Macro-level national planning, however, is not designed to address the special circumstances and requirements of each facility at the local level, where services are delivered.

To address the local context in terms of staff deployment and utilization, it is essential to engage the provincial authorities in HRH planning and management and to ensure their capacity and commitment to resolve challenges arising in the provision of health services. In keeping with the strategic direction and parameters of the national plan, the provincial plan should ensure availability of essential health staff based on local service priorities, actual utilization of services in the respective facilities, and other strategic considerations, such as support for remote and hard-to-reach areas. In formulating local solutions to improve workload and productivity, the provinces could take into account the location of facilities, the demographics of the catchment population, the particular living and working conditions, the optimal utilization of the staff, the required staff mix, and the arrangements for adequate staff supervision.

## OBJECTIVES

This article aims to describe the process of developing the provincial health workforce development plan, including the difficulties and challenges encountered and the lesson learned, based on a case study of support given to Bolikhamxay (BLX), a central province in Lao PDR.

### Plan-making process

#### Situational analysis

The process commenced with a series of workshops with the provincial health department (PHD) and district health officers to assess the current situation, including staff availability, distribution, and deployment of health staff at provincial, district, and HC levels. The initial review revealed inconsistencies with the information available at the national level, as well as out of date records and inaccuracies at both the provincial and district levels. To address the HRH information deficiency, field visits were arranged to all districts and selected HCs in order to complement and verify HRH information against staff lists used at the PHD. The discussions held at the district level also enabled a more detailed analysis of the priority staffing difficulties and constraints regarding access to quality services from the perspective of the local staff and leaders.

#### Projection of staff needs

Following the verification of the data, a provincial HRH projection model of BLX Province was developed based on the review of actual existing staff against the national staffing norms for the province, district, and HC levels. The provincial projection model indicated staff needs for the next 10 years (2015–2025), the gap between staff needs and existing staff, requirement of annual staff deployment (taking into account staff loss due to different reasons, including retirement), and workload and productivity of district and HC staff.

#### Identification and prioritization of solutions

A workshop with PHD and district health managers was convened to present the results of the situational analysis and the implications for production, recruitment, and retention emerging from the PHD projection model, including associated costs. In this workshop, different strategic HRH interventions for resolving key constraints and HRH difficulties aimed at improving service delivery were considered and prioritized. Attention was given to variability in the number of staff deployed and utilized at the different facilities and the possibilities for improving the HRH situation through various interventions, such as reallocation and/or rotation among staff; task shifting, improvement of HRH management at district and provincial levels; and upgrading staff through short-term, mid-term, and long-term training. As a result of this workshop, which involved the participation of central, provincial, and district level focal persons, a framework for formulation of a provincial health workforce development plan was adopted.

### Health workforce development plan of Bolikhamxay province

#### Overall staffing requirement

**Figure 1.**
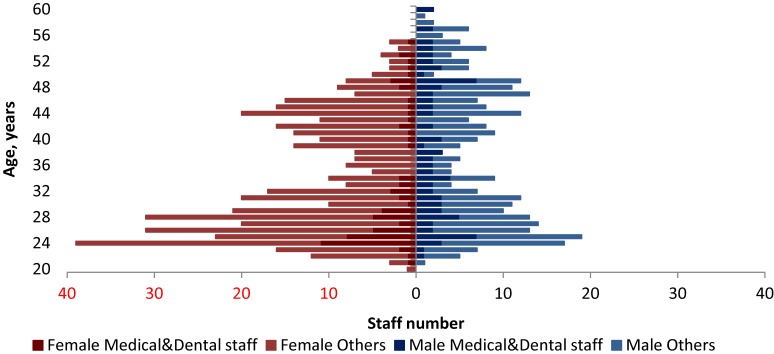
Age distribution by profession and gender.

At present, the BLX province employs 751 staff; of these, 24.5% are employed at the province level, 48.3% at district level, and 27.2% at the HC level (Table 1). According to the 2013 national HRH annual report of the DHP, BLX province had 20 staff per 10 000 population, which was lower than the national average. Age distribution by professions and gender was sown in Figure 1 indicating shortage of clinical staffs, particularly in the age group 30–40 years old, to provide supervision, guidance and support to junior staff in next 20 years.

**Table 1.** Existing health staff in 2014

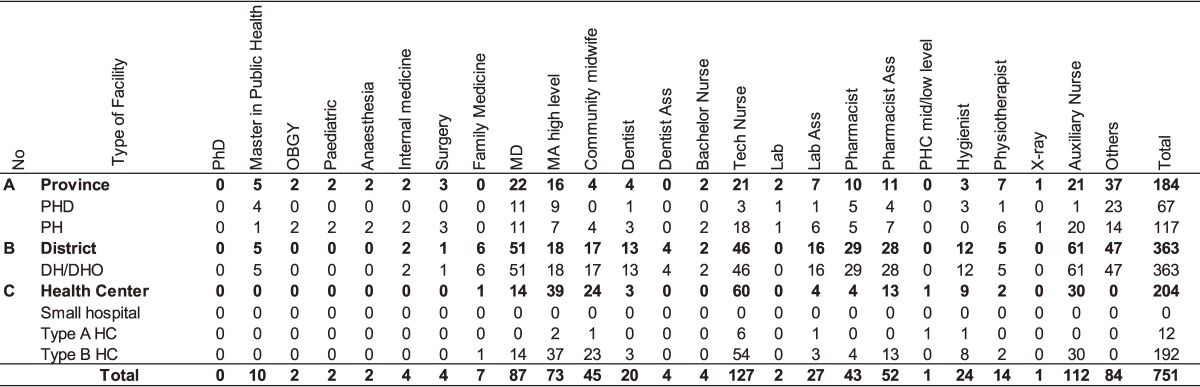

DH, district hospital; DHO, district health office; HC, health center; OBGY, obstetrics and gynecology; MD, medical doctor; MA, medical assistant; Dentist Ass, dentist assistant; Lab, laboratory technician; Lab Ass, laboratory assistant; PHC, primary health care; PH, provincial hospital; PhD, doctor of philosophy; PHD, provincial health department.

Based on the staffing assumptions set by the national staff projection model, BLX Province requires 998 HWs to meet the staffing targets to be achieved during the projection period (2015–2024) (Table 2). Hence, the total staffing gap of BLX for the projection period is 247 HWs, implying an increase of 32.9% in relation to the existing staff over the next 10 years. The projected distribution of the additional staff amounts to 81 health staff (32.9%) for the province level, 129 health staff (52.2%) for the district level, and 37 health staff (15.0%) for the HC level (Table 3). Based on the projected distribution, the proportion of staff will be slightly changed to 26.6% for provincial level, 49.3% for district level, and 24.1% for the HC level in comparison with staffing in 2015. The staffing requirements in the different disciplines and health facilities are presented in Table 3. The estimate of additional staff required from 2015 to 2024 includes around 11 officers with post-graduate public health training; 15 specialists, including family medicine (FM); 30 midwifes; 165 high-level nurses; 79 hygienists; and 8 medical imaging specialists (Table 3).

**Table 2.** Staffing needs to meet staffing assumption of the MOH

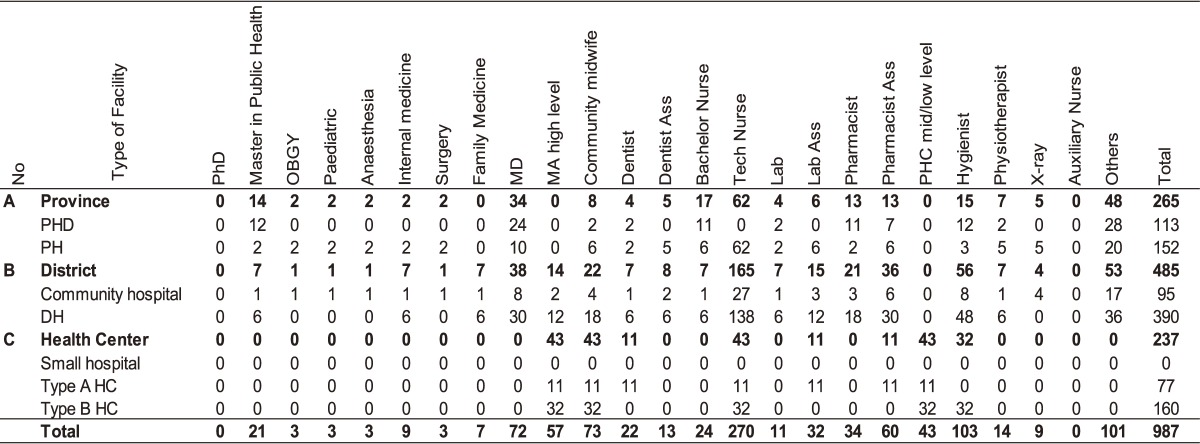

DH, district hospital; DHO, district health office; HC, health center; OBGY, obstetrics and gynecology; MD, medical doctor; MA, medical assistant; Dentist Ass, dentist assistant; Lab, laboratory technician; Lab Ass, laboratory assistant; PHC, primary health care; PH, provincial hospital; PhD, doctor of philosophy; PHD, provincial health department.

**Table 3.** Gap between staffing needs and existing staff by 2024

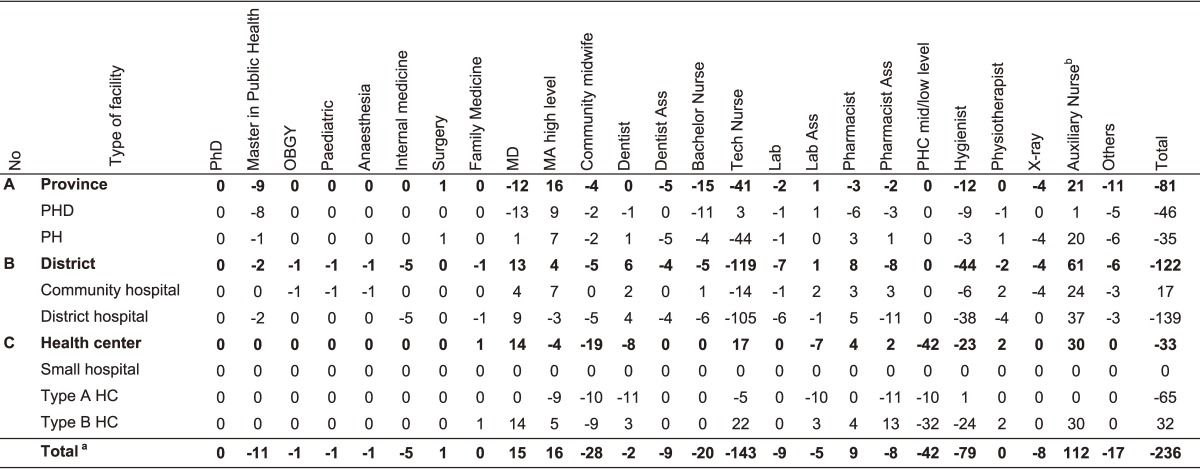

DH, district hospital; DHO, district health office; HC, health center; OBGY, obstetrics and gynecology; MD, medical doctor; MA, medical assistant; Dentist Ass, dentist assistant; Lab, laboratory technician; Lab Ass, laboratory assistant; PHC, primary health care; PH, provincial hospital; PhD, doctor of philosophy; PHD, provincial health department.

^a^The estimation of gap is based on target for additional staff not including loss of staff due to attrition (see Table 4).

^b^The auxiliary nurse cannot substitute for other categories of staff. When retire will not be replaced; therefore, not included in estimation of the gap. If the total staff requirement based on staffing gap (−236) plus estimated attrition (−115) and surplus auxiliary nurse (−81) and pharmacists (−8) amount to −440.

The projection model estimates take into account attrition of staff due to retirement and other reasons. Table 4 shows projected number of staff by category reaching retirement age from 2015–2024. Based on the projection of the age of staff, 115 health staff will reach retirement age (55 years old for women and 60 years old for men). From 2015 to 2024, BLX Province should receive a recruitment quota of approximately 451 health staff to meet the gap; on average, the PHD should be allocated an additional 44 health staff annually (Table 5).

**Table 4.** Estimated number of staff reaching retirement age from 2015–2024

No	Staff category	Estimated number of retirement											% of total

		2015	2016	2017	2018	2019	2020	2021	2022	2023	2024	Total	
1	PhD											0	0.0
2	Master in Public Health							1		1		2	20.0
3	OBGY									1		1	50.0
4	Paediatric						1					1	50.0
5	Anaesthesia											0	0.0
6	Internal medicine											0	0.0
7	Surgery								1			1	25.0
8	Family Medicine											0	0.0
9	MD		2	2	1	3	2	2	1	2	2	17	19.5
10	MA high level	1		2	2	1	6	3		2	2	19	26.0
11	Community midwife					1				1	2	4	8.9
12	Dentist											0	0.0
13	Dentist Ass	1				1						2	50.0
14	Bachelor Nurse						1			1		2	50.0
15	Tech Nurse							1	1		1	3	2.4
16	Lab							1				1	50.0
17	Lab Ass			2			1		2			5	18.5
18	Pharmacist					1						1	2.3
19	Pharmacist Ass				2		2					4	7.7
20	PHC mid/low level											0	0.0
21	Hygienist											0	0.0
22	Physiotherapist					1				1	1	3	21.4
23	X-ray											0	0.0
24	Auxiliary Nurse^a^		2	1	1	1	1	3	4	10	8	31	27.7
25	Others	1	2	2		1	2	2	4	2	2	18	21.4

	Total	3	6	9	6	10	16	13	13	21	18	115	

OBGY, obstetrics and gynecology; MD, medical doctor; MA, medical assistant; Dentist Ass, dentist assistant; Lab, laboratory technician; Lab Ass, laboratory assistant; PHC, primary health care; PhD, doctor of philosophy.

Note: retirement age of 60 ys for male and 55 ys for female.

^a^The auxiliary nurse cannot substitute for other categories of staff. When retire will not be replaced; therefore, not included in estimation of the gap. If the total staff requirement based on staffing gap (−236) plus estimated attrition (−115) and surplus auxiliary nurse (−81) and pharmacists (−8) amount to −440.

**Table 5.** Expected requirement for recruitment by year from 2015–2024

No	Staff category	Annual recruitment										Total

		2015	2016	2017	2018	2019	2020	2021	2022	2023	2024	
1	PhD	0	0	0	0	0	0	0	0	0	0	0
2	Master in Public Health	1	1	1	1	1	1	1	1	1	1	13
3	OBGY	0	0	0	0	0	0	0	0	0	0	2
4	Paediatric	0	0	0	0	0	0	0	0	0	0	2
5	Anaesthesia	0	0	0	0	0	0	0	0	0	0	1
6	Internal medicine	1	1	1	1	1	1	1	1	1	1	5
7	Surgery	0	0	0	0	0	0	0	0	0	0	0
8	Family Medicine	0	0	0	0	0	0	0	0	0	0	0
9	MD	0	0	0	0	0	0	0	0	0	0	2
10	MA high level	0	0	0	0	0	0	0	0	0	0	3
11	Community midwife	3	3	3	3	3	3	3	3	3	3	32
12	Dentist	0	0	0	0	0	0	0	0	0	0	2
13	Dentist Ass	1	1	1	1	1	1	1	1	1	1	11
14	Bachelor Nurse	2	2	2	2	2	2	2	2	2	2	22
15	Tech Nurse	15	15	15	15	15	15	15	15	15	15	146
16	Lab	1	1	1	1	1	1	1	1	1	1	10
17	Lab Ass	1	1	1	1	1	1	1	1	1	1	10
18	Pharmacist	0	0	0	0	0	0	0	0	0	0	0
19	Pharmacist Ass	1	1	1	1	1	1	1	1	1	1	12
20	PHC mid/low level	4	4	4	4	4	4	4	4	4	4	42
21	Hygienist	8	8	8	8	8	8	8	8	8	8	79
22	Physiotherapist	0	0	0	0	0	0	0	0	0	0	3
23	X-ray	1	1	1	1	1	1	1	1	1	1	8
24	Auxiliary Nurse	0	0	0	0	0	0	0	0	0	0	0
25	Others	3	3	3	3	3	3	3	3	3	3	35

	**Total**	**44**	**44**	**44**	**44**	**44**	**44**	**44**	**44**	**44**	**44**	**440**

OBGY, obstetrics and gynecology; MD, medical doctor; MA, medical assistant; Dentist Ass, dentist assistant; Lab, laboratory technician; Lab Ass, laboratory assistant; PHC, primary health care; PhD, doctor of philosophy.

Note: The expected requirement for recruitment is based on average number per year over the next 10 years. In practice certain category such as community midwife and MA high level will be recruited earlier to meet service priority.

Generally, BLX Province seems to have enough medical doctors (MD) and medical assistants (MA) (at least 12 MD and 17 MA more than the projected number), with the exception of Viengthong district, where the plan to upgrade the district hospital (DH) to a higher-level community hospital will require additional medical practitioners.

The increase of staff numbers will require an increase in salary and allowances for staff. It is estimated that, by 2024, the annual salary of staff for BLX PHD will be about 3.1 million United States dollars, increased from 2.4 million United States dollars in 2015, implying an increase of approximately 2.6% to 3.2% per year (data not shown). The estimation is based on an assumption of no change in salary scale over the projected period.

#### Targets for staff deployment priorities

• Deployment of midwives in all health centers nationwide is among the top priorities of the MOH. The PHD aims to achieve the target of at least one community midwife at each health center by 2016. Currently, only 26 HCs employ a midwife, implying a deficit of 25 HCs that require a midwife (Table 6). Of these, 14 HCs (58.3%) are not able to provide normal assisted delivery service due to a lack of qualified staff with essential skills in assisting normal delivery, such as MDs, MAs, and registered nurses. Six districts, with the exception of Xaychamphone, have high numbers of HCs without midwives and should be given priority in allocation of graduating community midwives in 2015.
• In line with the standards set by the MOH, the PHD aims to deploy a team of 5–7 HWs in Type B HCs and around 10 HWs in Type A HCs, which will perform as small hospitals.^6^ The current staff number at the HC level in BLX Province shows that 15 of the 41 HCs (36.6%) have fewer than 5 HWs; of these, 4 HCs (9.8%) have only three or even fewer health staff. This major gap should to be addressed during the next 10 years.
• Beyond the total size of the team, it is essential to ensure an adequate skill mix, including capacity to provide basic curative care as well as adequate capacity for managing the team. To this end, it is essential to ensure that each HC has at least one or two high-level staff (MD, MA, and/or registered nurse). There are currently 24 HCs (58.5%) without capacity to provide basic curative care (ie, without staff that have high-level qualifications).
• At the district level, it is noted that six of seven districts have a FM doctor, and, of the entire districts in BLX Province, seven have FM specialists. The PHD will aim to supply doctors with FM qualifications to all the districts within the next 3 years. Currently, only Thaphabath DH is without a FM specialist; however, it has one internal medicine specialist.
• Priority will also be given to posting at least one staff member with a master’s of public health (MPH) degree in each district. Currently, Thaphabath district and Xaychamphone have no officers with an MPH degree, indicating a major need for this category of staff during the next 3–5 years.
• Based on the projection model, the BLX provincial hospital (PH) needs more registered nurses to meet its staffing standard. There are currently 20 nurses in the PH; however, approximately 68 registered nurses are needed by 2024, so the gap to be bridged is 48 registered nurses.
• Many services, such as ophthalmology and ear, nose, and throat (ENT), are provided by general practitioners, including MDs and nurses. Based on the service demand, the PH needs four specialists in medical imaging (two ultrasound specialists and two X-ray specialists); currently, there is only one X-ray practitioner in the hospital. In addition to this, the new PH will be built in the near future; therefore, projection of staffing needs of the hospital should be re-assessed in accordance with the increased number of beds and types of health services.

**Table 6.** Shortage and skill mix of health center staff during 2014

	District	Total HC number	Total staff number in 2014	HC without clinician	HC without midwife	HC without delivery service
1	Khamkeuth	9	46	3	6	2
2	Thaphabath	5	22	3	4	3
3	Bolikhan	6	31	4	4	2
4	Pakkading	5	30	2	3	2
5	Pakxan	6	33	2	3	1
6	Viengthong	6	24	6	4	4
7	Xaychamphone	4	18	4	1	0

	Grand total	41	204	24	25	14

HC, health center.

### Variation in utilization of services and productivity of staff

The PHD will monitor the level of health services activity in health facilities and document these services for review. In making allocation of staff to health facilities, the PHD will consider:

The level of health services activities performed by each facility, the catchment population benefiting from the services, and other factors, such as location availability of alternative services and other issues related to access.

#### District hospital and office

**Figure 2.**
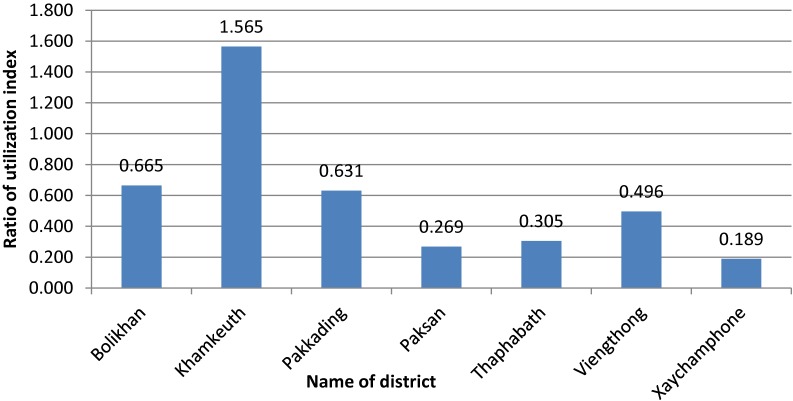
Utilization index of health service for district hospital and office during 2014. Utilization index = total number of weighted health service activities/total number of catchment population; health service activities including curative and preventive services (such as outpatient; inpatient; Diphtheria, Tetanus and Pertussis vaccine in children; tetanus toxoid vaccine in childbearing aged women; institutional delivery; antenatal care, and postnatal care).

**Figure 3.**
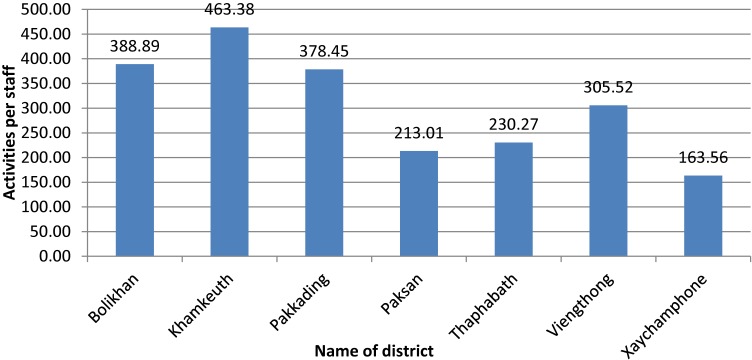
Average health service activities per staff of district hospital and office during 2014. Health service activities per staff = total number of weighted health service activities/total staff at district level.

As shown in Figure 2, the highest ratio of health services activities in relation to the size of the catchment population is found in Khamkeuth DH, while Xaychamphone has the lowest utilization. Low rates of utilization are also found in Pakxan and Thaphabath. There is no DH in Pakxan. Figure 3 to Figure 5 show the ratio of HWs to health service activities, the ratio of health staff to the catchment population and level of productivity of district hospitals.

**Figure 4.**
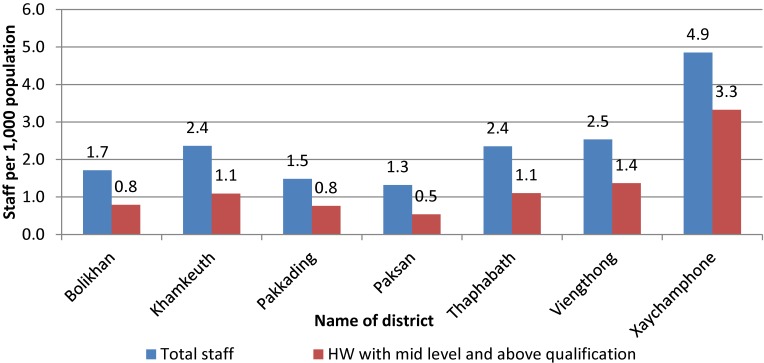
Ratio of district staff per 1000 catchment population during 2014. District staff are combination of district hospital staff and district health office staff; ratio of district staff = total district staff * 1000/total number of catchment population. HW, health worker.

**Figure 5.**
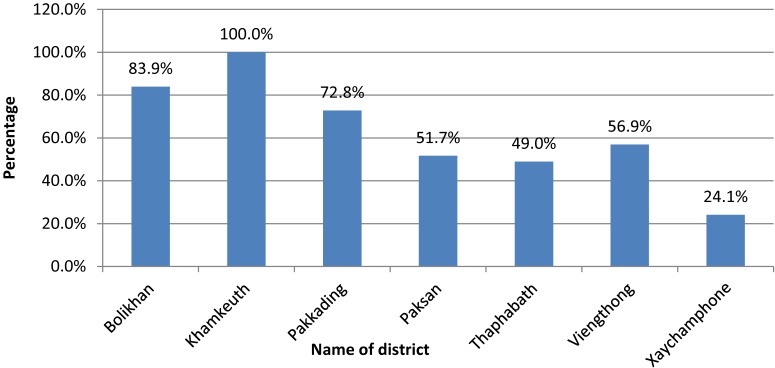
Productivity of district staff during 2014. Productivity = total number of weighted health service activities/total salary of staff at district level; District with the highest productivity score is used as reference to calculate percentage.

Based on the variation in average number of activities per staff (workload), utilization of services, and availability of staff, the PHD will need to pay particular attention to the less active facilities (Xaychamphone, Thaphabath, and Viengthong districts) to determine their role and ways to improve their utilization rates and staff performance. The PHD plans to upgrade Viengthong DH to a community hospital, which will require further allocation of staff and provisions for upgrading existing staff.

#### Health centers

**Figure 6.**
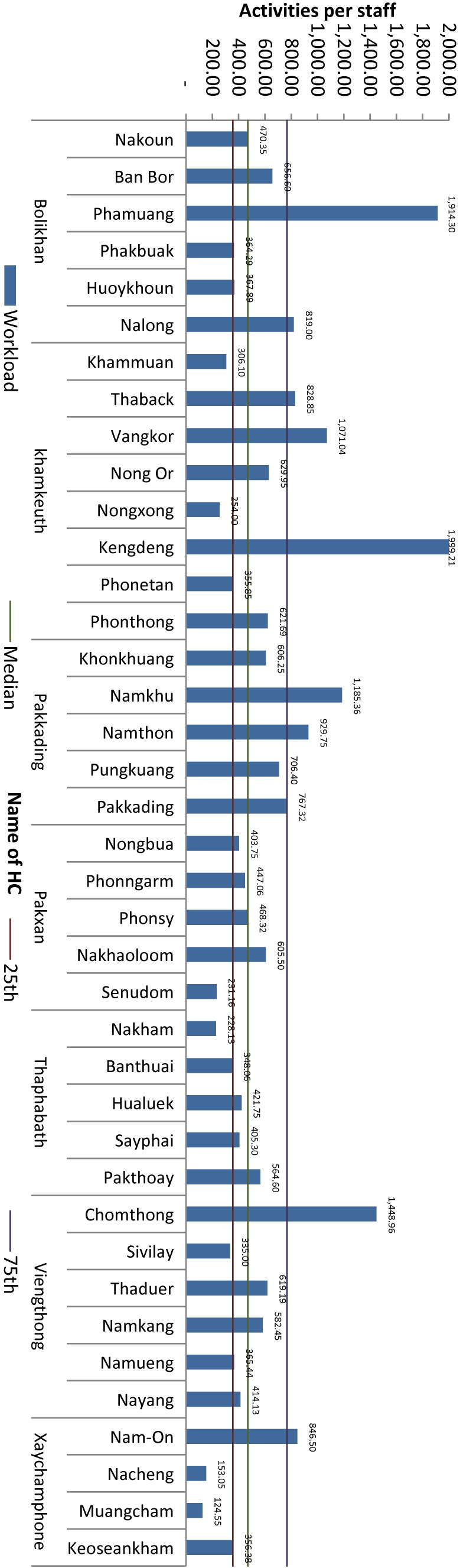
Average health service activities per health center staff during 2014. Health service activities per staff = total number of weighted health service activities/total number of health center staff. HC, health center.

Analysis of services provided in HCs in relation to the number of staff members and the catchment population enables estimation of staff workload and utilization of the HC by the local community (Figure 6). Again, variability among the HCs will trigger a review of probable reasons and identification of interventions to improve the demand for services and staff productivity. The PHD will give priority attention to HCs with low utilization and determine priorities for allocation of staff over the planning period 2016–2020.

### Recommendations

The provincial health department, in close coordination with the MOH and other authorities at provincial and district levels, will build capacity for and promote effective management of human resources to:

• Improve staff performance and motivation through regular systematic supportive supervision programs;
• Ensure an adequate mix of skills required to perform agreed-upon services at different health facilities and services;
• Improve the competence of health personnel by determining and implementing affordable and sustainable approaches to training and professional development of staff in order to strengthen their ability to deliver quality services and demonstrate high-level professional commitment and ethical practice;
• Allocate fellowships and scholarships based on training needs assessment for upgrading low- and mid-level staff to high-level staff; and training sufficient midwives and specialists in different disciplines; and
• Provide on-the-job and in-service trainings to improve knowledge and clinical skills of health staff at district and HC levels.

### Lesson learned

The implementation of the staffing standards at the local level requires local analyses and engagement of local health authorities to validate the HRH information, perform case-by-case review, and resolve local issues. The formulation of provincial workforce development plans requires strong collaboration with the national authorities and development partners to ensure adequate support and resourcing. Last but not least, lessons derived from this initial intervention will enable the MOH to extend and scale the effort to include all 18 provinces in Lao PDR and to attain significant improvement in the utilization, productivity, and performance of the health workforce.
